# Thyroid disease in insulin-treated patients with type 2 diabetes: a retrospective study

**DOI:** 10.1186/1756-6614-7-2

**Published:** 2014-03-01

**Authors:** Valerie Witting, Dominik Bergis, Dilek Sadet, Klaus Badenhoop

**Affiliations:** 1 Department of Internal Medicine 1, Division of Endocrinology & Metabolism, Goethe-University Hospital, Theodor-Stern-Kai 7, 60590 Frankfurt am Main, Germany

**Keywords:** Thyroid disease, Diabetes mellitus, Coexistence, Outcome, Insulin therapy

## Abstract

**Background:**

Diabetes mellitus and thyroid diseases frequently coexist. In order to evaluate how thyroid disorders interfere with glycemic control, we analysed insulin-treated type 2 diabetes patients with thyroid disease.

**Methods:**

Diabetes patients (n = 1.957) were retrospectively investigated. We focused on type 2 diabetes patients who had been admitted for insulin-treatment and diagnosed thyroid diseases (n = 328). Patients were divided into three groups according to thyroid disease manifestation in relation to diabetes onset: prior to (group 1), same year (group 2) and thyroid disease following diabetes (group 3).

**Results:**

Out of all diabetes patients 27.3% had a thyroid disorder with more women (62.2%) being affected (p < 0.001). Thyroid disease was predominantly diagnosed after diabetes onset. Patients with type 2 diabetes and prior appearance of thyroid disease required insulin therapy significantly earlier (median insulin-free period: 2.5 yrs; Q_1_ = 0.0, Q_3_ = 8.25) compared to patients who had thyroid dysfunction after diabetes onset (median insulin-free period: 8.0 yrs; Q_1_ = 3.0, Q_3_ = 12.0; p < 0.001). Age at diabetes onset correlated with insulin-free period (p < 0.001).

**Conclusions:**

Thyroid disease may be a marker of a distinct metabolic trait in type 2 diabetes potentially requiring earlier insulin treatment.

## Introduction

Thyroid diseases have been described to be more common in diabetes than expected. In the general population their prevalence range between 6.6% in the UK [1] and 5.9% in the US National Health and Nutrition Examination Survey (NHANES III) [2]. In contrast, diabetes mellitus shows a higher prevalence - 10.8% in the community and up to 13.4% in a hospital diabetic clinic [3,4].

These endocrinopathies influence each other in multiple ways. Poorly controlled diabetes mellitus may affect thyroid metabolism as uncontrolled hyperglycemia alters plasma triiodothyronine (T3) and in part thyroxine (T4) levels [5]. Hyperinsulinaemia can also be associated with goitre as well as with thyroid nodules [6,7]. Conversely, hyperthyroidism can aggravate glycemia by a stimulation of glucose absorption, glycogenolysis and hepatic glucose production [8] as well as enhanced insulin resistance [9] and shortened half-life of circulating insulin concentrations [10]. Reverse effects have been observed in patients with hypothyroid metabolism [11].

These interactions need to be understood for clinical management. The present study analysed the impact of thyroid diseases on glycemic control comparing insulin-treated type 2 diabetes patients with thyroid disorder manifestation prior to, concomitant with and following diabetes onset.

### Subjects and methods

#### Subjects

We retrospectively evaluated 1.957 diabetes patients (843 females, 1.114 males) who were admitted to the Department of Endocrinology & Diabetes at the University Hospital Frankfurt am Main, Germany, between January 1, 2002, and December 31, 2010. The retrospective study on anonymized patient data was performed in compliance with the Helsinki declaration.

Patients were classified as having coexistent thyroid dysfunctions based on information on autoantibodies against thyroid peroxidase (anti-TPO), thyroglobulin (anti-Tg) and thyrotropin receptor (TRAb), thyroid function (serum free triiodothyronine (fT3), free thyroxine (fT4), thyroid-stimulating hormone (TSH)), thyroid volume (sonography) as well as thyroid disease treatment for individual diagnoses according to the following criteria:

Hypothyroidism – including subclinical – defined as serum TSH levels greater than the corresponding reference range associated with normal or decreased serum fT4 and fT3 values. Hyperthyroidism – including subclinical – diagnosed by suppressed serum TSH levels with normal or increased concentrations of fT4 and fT3. Goitre defined as thyroid enlargement with a total glandular volume exceeding 18 ml in women and 25 ml in men, corresponding to mean thyroid volume +3 SD in iodine-sufficient populations [12]. This definition has also been applied in other European studies on the epidemiology of thyroid disorders [13]. Since our patients grew up during iodine deficient periods, we decided to apply this definition bearing in mind that the standards to define normal thyroid volumes are changing [14,15]. Hashimoto’s thyroiditis confirmed by the presence of elevated anti-TPO or anti-Tg antibodies associated with primary hypothyroidism and, if available, by cytological evidence of lymphocytic infiltration and an ultrasound image of hypoechogenicity. Graves’ disease was diagnosed by hyperthyroidism with either diffuse goitre, positive TRAb or the presence of ophthalmopathy, but not all of our patients were thyroid antibody tested. For statistical analyses Hashimoto’s thyroiditis and Graves’ disease were subsumed as autoimmune thyroid disease (AITD).

For closer investigations we focused on insulin-treated type 2 diabetes patients (diagnosed based on WHO criteria) with coexistent thyroid dysfunctions (n = 328) and additional available parameters: body height and weight, date of insulin initiation and titres of autoantibodies against glutamate decarboxylase (anti-GAD). These patients were divided into three groups: thyroid disease manifestation prior to (**group 1**), same year (**group 2**) and following diabetes mellitus onset (**group 3**). Due to incomplete parameters in some patients, the total number of patients varies in some comparisons (cf. Results).

#### Statistical analysis

The Statistical analysis was carried out by software SPSS version 11.5 and Bias Statistical package version 10. The Kolmogorov test was applied to test for normal distribution of the data. Due to a missing normal distribution, two groups of quantitative variables were calculated and compared using Mann-Whitney U test, whereas comparison of more than two groups was performed by the Kruskal-Wallis test. Data on quantitative parameters were expressed as median and quartilesQ_1_ (first quartile) and Q_3_ (third quartile). Data on qualitative parameters were expressed as percentages as well as absolute values, and were compared by the chi-square (χ^2^) test or the Fisher’s exact test. Statistical dependence was measured by Spearman's rank correlation coefficient (r_s_), its degree of influence was interpreted by the coefficient of determination (r_s_^2^). The level of statistical significance was set at p < 0.05.

## Results

### Study population

Between January 1, 2002, and December 31, 2010, a total of 1.957 diabetes patients were admitted as inpatients to the Department of Endocrinology & Diabetes at the University Hospital Frankfurt am Main, Germany. In 535 (27.3%) diabetes patients a thyroid disease had been diagnosed: hypothyroidism (n = 349), hyperthyroidism (n = 194), Graves’ disease (n = 37), Hashimoto’s thyroiditis (n = 145), goitre (n = 221), autonomously functioning thyroid (AFT, n = 22), thyroid carcinoma (n = 5). 124 (23.2%) of them did not receive any thyroid treatment so far. A clear majority (n = 225) developed their thyroid disease after diabetes onset, whereas due to missing information 212 patients could not be classified in this regard. With advancing age manifestations of thyroid disease steadily increased up to a peak in the seventh decade. In 58.4% of the patients thyroid disease had been diagnosed after the age of 50.

Diabetes patients with coexisting thyroid disease consisted of 202 men (37.8%) and 333 women (62.2%) whilst those without thyroid disorders presented a statistically significantly higher percentage of men (64.1% men (n = 912), 35.9% women (n = 510), p < 0.001). Due to the large number of patients without thyroid disease, 560 were randomly selected for further analysis resulting in 559 finally included in the study. A comparison of median HbA1c values was performed in type 2 diabetes patients. Patients with thyroid dysfunction had significantly lower median levels of HbA1c (7.2%; Q_1_ = 6.5, Q_3_ = 8.2) compared to those without thyroid dysfunction (7.5%; Q_1_ = 6.6, Q_3_ = 8.9; p < 0.01) and this difference was not explained by gender.

### Insulin-treated type 2 diabetes patients with thyroid dysfunction

Insulin-treated type 2 diabetes patients with coexistent thyroid dysfunctions (n = 328) were divided into three groups according to their sequence of diagnoses when known (140 patients could not be classified resulting in 188 patients for following analyses): 133 (70.7%) had thyroid disease diagnosed after diabetes onset (group 3) whereas less patients developed thyroid diseases prior to (group 1; n = 46; 24.5%) or within the same year (group 2; n = 9; 4.8%) of diabetes onset.

Sex ratio and the medians of age, age at diabetes and thyroid disease onset, BMI (body mass index) and insulin-free period in groups 1 to 3 and in the total cohort (n = 188) are listed in Table 1. Some data was partially missing: body height in 6 patients, date of insulin initiation in 23 patients.

**Table 1:** Sex ratio and median values of quantitative parameters (age, age at diabetes and thyroid disease onset, BMI, insulin-free period)

	**Group 1**	**Group 2**	**Group 3**	**Total**	**Statistical significance**
Sex - male/female	8/38	5/4	61/72	74/114	p < 0.01
Age [yrs]	61.5	54.0	60.0	61.0	ns
Age at diabetesonset	53.0	60.0	49.0	50.5	p < 0.01
Age at thyroid disease onset	43.0	60.0	62.0	60.0	p < 0.001
BMI [kg/m^2^]	28.7	28.1	30.6	30.3	ns
Insulin-free period [yrs]	2.5	0.0	8.0	7.0	p < 0.001

Group definition: Thyroid disease diagnosed prior to (group 1), within the same year (group 2) or after (group 3) diabetes onset.

BMI: Body Mass Index.

Statistically significant differences were observed for age at diabetes onset (p < 0.01): Patients of group 1 (p < 0.01) and 2 (p < 0.05) were significantly older at diabetes onset when compared to patients of group 3. Group 2 comprised the eldest patients. Contrasting results were found for age at thyroid disease onset: Patients with thyroid disease prior to diabetes onset (group 1) were significantly younger at thyroid disease onset than patients with concomitant (group 2; p < 0.01) or subsequent manifestation of thyroid disease (group 3; p < 0.001). BMI did not differ statistically significantly among the groups with high median values between 28.1 kg/m^2^ and 30.6 kg/m^2^.

As a measure of individual beta-cell reserve for metabolic control, the time between diabetes onset and initiation of insulin substitution, i.e. the insulin-free period, was investigated in the different groups. With a median value of 8.0 years (Q_1_ = 3.0, Q_3_ = 12.0) patients with thyroid disease diagnosed after diabetes mellitus (group 3) required insulin therapy significantly later when compared to patients with thyroid disease diagnosed prior to (group 1; p < 0.001) or within the same year (group 2; p < 0.001) of diabetes onset. A comparison of group 1 (2.5 yrs; Q_1_ = 0.0, Q_3_ = 8.25) and 2 (0.0 yrs; Q_1_ = 0.0, Q_3_ = 0.75) also showed a significant difference (p < 0.05). Patients of group 3 developed thyroid diseases on average only after the initiation of insulin treatment.

**Figure 1:**
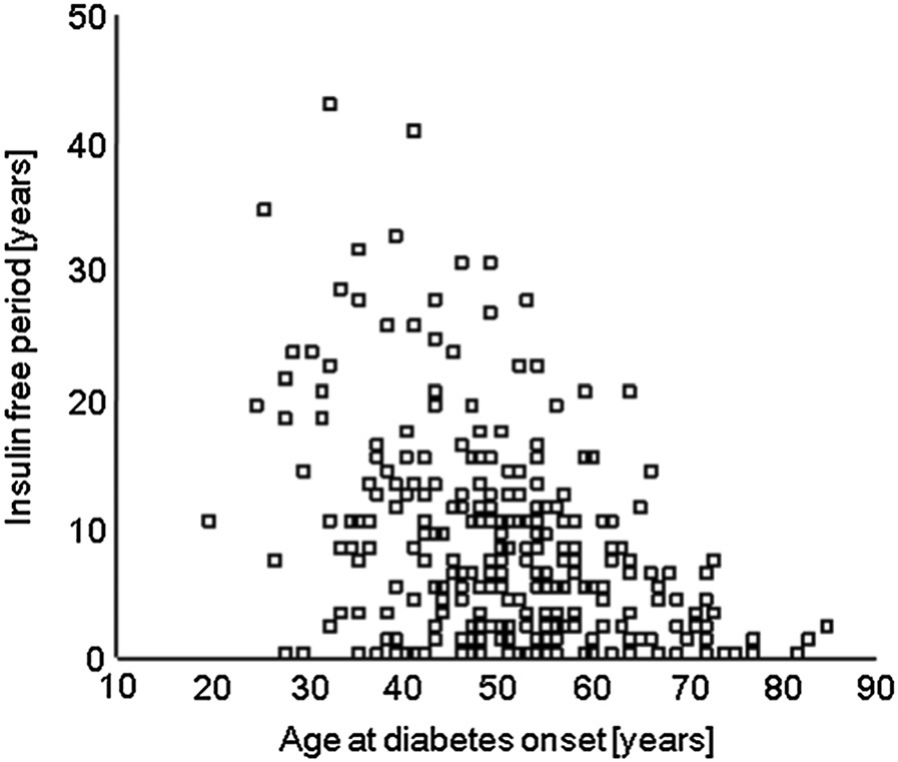
Correlation between age at diabetes onset and insulin-free period (n = 283).

An investigation of possible influencing factors showed no correlation between BMI and insulin-free period (p > 0.05). However, a clear negative correlation was observed between age at diabetes onset and insulin-free period (p < 0.001; r_s_ = -0.364, r_s_^2^ = 13.3%, Figure 1). The older the patient the sooner insulin treatment was started.

Frequencies of positive antibodies (anti-GAD, anti-TPO, anti-Tg), thyroid diseases, thyroid treatment (including partial and complete thyroidectomy, radioiodine and oral medication) and Levothyroxine supplementation in particular in groups 1 to 3 and in the total cohort (n = 188) are detailed in Table 2. Data was partially missing: titres of anti-GAD in 144 patients, titres of anti-TPO in 64 patients, titres of anti-Tg in 65 patients.

**Table 2:** Frequencies of positive antibodies (anti-GAD, anti-TPO, anti-Tg), thyroid diseases, thyroid treatment and Levothyroxine supplementation

	**Group 1**	**Group 2**	**Group 3**	**Total**	**Statistical significance**
Anti – GAD	0 (0.0%)	0 (0.0%)	1 (3.0%)	1 (2.3%)	ns
Anti – TPO	2 (10.0%)	3 (50.0%)	24 (24.5%)	29 (23.4%)	ns
Anti – Tg	2 (10.0%)	2 (33.3%)	18 (18.6%)	22 (17.9%)	ns
Hypothyroidism	13 (28.3%)	4 (44.4%)	54 (40.6%)	71 (37.8%)	ns
Hyperthyroidism	8 (17.4%)	3 (33.3%)	55 (41.4%)	66 (35.1%)	p < 0.05
AITD	13 (28.3%)	4 (44.4%)	28 (21.1%)	45 (23.9%)	ns
Goitre	28 (60.9%)	1 (11.1%)	51 (38.3%)	80 (42.6%)	p < 0.01
With AITD	5 (10.9%)	0 (0.0%)	6 (4.5%)	11 (5.9%)	ns
Without AITD	23 (50.0%)	1 (11.1%)	45 (33.8%)	69 (36.7%)	p < 0.05
AFT	2 (4.4%)	0 (0.0%)	8 (6.1%)	10 (5.3%)	ns
Carcinoma	0 (0.0%)	1 (11.1%)	3 (2.3%)	4 (2.1%)	ns
Thyroid treatment	45 (97.8%)	6 (66.7%)	90 (67.7%)	141 (75.0%)	p < 0.001
Levothyroxine	41 (89.1%)	5 (55.6%)	69 (51.9%)	115 (61.2%)	p < 0.001

Group definition: Thyroid disease diagnosed prior to (group 1), within the same year (group 2) or after (group 3) diabetes onset.

Thyroid treatment: Including partial and complete thyroidectomy, radioiodine and oral medication.

anti-GAD: autoantibodies against glutamate decarboxylase; anti-TPO: autoantibodies against thyroid peroxidase; anti-Tg: autoantibodies against thyroglobulin; AITD: autoimmune thyroid disease; AFT: autonomously functioning thyroid.

Only one patient – belonging to group 3 – was found to be anti-GAD-positive, whereas no patient of group 1 or 2 was anti-GAD-positive. No significant differences were observed for positivity of thyroid autoantibodies (anti-TPO, anti-Tg). However, patients in group 2 had the highest frequency of positive results for anti-TPO as well as anti-Tg.

We then focused on primary thyroid dysfunction. Statistically significant differences were found in the prevalence of hyperthyroidism among individual groups (p < 0.05). The prevalence of hyperthyroidism was highest in group 3 (41.4%) being significantly more frequent than in group 1 (17.4%; p < 0.01). With advancing age the prevalence of hyperthyroidism increased more compared to the prevalence of other thyroid disorders. Whereas hypothyroidism was diagnosed in 40.8% after the age of 59, this applied to 59.0% of the patients with hyperthyroidism. After excluding all patients with hyperthyroidism from the analysis, the difference regarding the insulin-free period between the groups remained statistically significant (p = 0.002). Furthermore, patients in group 1 suffered most frequently from goitre, significantly fewer were found in group 2 (p < 0.01) and group 3 (p < 0.01).

The small size of group 2 (n = 9) makes it difficult to compare it with the other groups, but we decided to keep this group in the retrospective analysis, since it comprises the clinical relevant co-diagnosed comorbidity in contrast to either chronic conditions known for longer times or later diagnosed disease that needs to be born in mind as potential to be screened for. We compared all our results before and after exclusion of group 2 from the analysis. No difference was found except in the comparison regarding frequency of hyperthyroidism, which showed a statistical significance of p < 0.01 instead of p < 0.05.

## Discussion

Diabetes patients run a higher risk of developing thyroid disorders [3,4,16,17]. In both the general population [1] and diabetes patients [3] thyroid diseases were reported to be more common in women than in men. Our results confirm this in German inpatient diabetes patients, as women (62.2%) were more frequently affected than men (37.8%).

By metabolic comparison type 2 diabetes patients showed significantly lower HbA1c when they had thyroid disease (7.2% vs. 7.5%; p < 0.01). This difference may mark the cohort with thyroid comorbidity to be distinct but missing information on compliance and dosage of thyroid as well as antidiabetic medication necessitate further studies to clarify this. However, other studies did not report any difference in HbA1c levels between type 2 diabetes patients with and without thyroid dysfunction [18-20]. In patients with type 1 diabetes Mohn et al. reported an association between symptomatic hypoglycemia and subclinical hypothyroidism without significant changes in HbA1c levels [16]. Thus, our results might not directly be attributable to thyroid comorbidity.

Whether thyroid disorders develop prior to or after diabetes may affect prognosis and metabolic control. In this study the majority of the diabetes patients developed their thyroid disease after diabetes onset. This can be explained by the increasing prevalence of thyroid disorders with advancing age as described in various studies [2,21] and confirmed by our results. Furthermore, following diabetes onset the patients are screened for thyroid disorders. However, our results underscore that diabetes patients are more susceptible to thyroid disorders [3,4,16,17].

In order to assess the different course of disease to insulin treatment of type 2 diabetes we analysed the insulin-free period dependent on the time of thyroid disease manifestation. A significantly earlier requirement of insulin therapy was observed in patients with thyroid disease onset prior to (group 1; 2.5 yrs; Q_1_ = 0.0, Q_3_ = 8.25) or within the same year (group 2; 0.0 yrs; Q_1_ = 0.0, Q_3_ = 0.75) of diabetes mellitus onset. This suggests that pre-existing thyroid disease may dysregulate metabolic control in newly diagnosed diabetes necessitating earlier insulin therapy. Maratou et al. [9] as well as Mohn et al. [11] already showed subclinical hypo- and hyperthyroidism may impact on glucose metabolism, an earlier requirement for insulin, however, has not been described before. Patients with concomitant appearance of the two endocrine disorders (group 2) required more often immediate insulin therapy despite their diagnosis of type 2 diabetes. A simultaneous disturbance of pancreatic and thyroidal metabolism deteriorates metabolic control and residual beta-cell function. Another study on diabetes patients without thyroid disorders showed treatment failure to sulfonylureas after a mean of 8.7 years in contrast to our group 1 and 2 patients who required insulin considerably earlier [22]. The insulin-free period in that study was not different to ours in group 3 (8.0 yrs; Q_1_ = 3.0, Q_3_ = 12.0). This confirms the difficulty of treating diabetes patients with coexistent thyroid disease. However, there are varying study criteria in the reports. We did not differentiate the antidiabetic treatments before insulin and had no information on whether insulin treatment was initiated after oral mono- or combination therapy.

In addition, there are further limitations: The high BMI ranging from 28.1 to 30.6 kg/m2 and the lack of anti-GAD autoantibody positivity in all groups make a diagnosis of type 2 diabetes mellitus very likely. However, latent autoimmune diabetes in adults (LADA) cannot be ruled out due to the lack of information on anti-GAD antibodies. Janka et al. reported that up to 10% of German type 2 diabetes patients may suffer from LADA [23] and patients with an autoimmune thyroid disease have an even higher risk of LADA. The earlier insulin requirement in our groups 1 and 2 may partly be an indication of underlying LADA. Also, in about 40% of our patients thyroid disease manifestation in relation to diabetes onset remained unclear. Although we cannot exclude that this introduces a selection bias, we regard it as a reflection of the fact that thyroid disorders are frequently neglected and time of onset more often forgotten than other disorders. Future studies will therefore need to document a full autoimmune status regarding diabetes mellitus and thyroid diseases.

BMI indicates insulin resistance and correlates inversely with beta-cell function [24,25]. We analysed the correlation between insulin-free period and BMI of our patients, but could not observe any. This result is in agreement with a French study in which insulin-dependent type 2 diabetes patients exhibited similar BMI to non-insulin-dependent patients [26]. However, age at diabetes onset and insulin-free period was inversely related: The later type 2 diabetes became manifest, the earlier patients needed insulin therapy, where about 13% variability of the insulin-free period is attributed to the age at diabetes onset. Our finding is compatible with another study in which the period of oral drug administration in patients who developed diabetes before the age of 50 was effectively longer than in patients with later onset of disease [22]. Older patients might have had undetected diabetes for a longer time span. Also elderly patients are more prone to develop a decline of beta-cell function. Thus, advanced age at diabetes onset in our groups 1 and 2 can partially be attributable for their earlier insulin requirement.

Previous studies suggest a relationship between positivity of islet cell antibodies and failure of oral antidiabetic drugs in type 2 diabetes where insulin deficiency explains therapy failure. Also thyroid antibodies were more frequent in non-responders [27,28]. In agreement with results of previous studies, we found positive anti-TPO and anti-Tg titres were most frequently in the group with immediate need of insulin treatment, although this was not significant due to the limited number of patients.

We confirm the increasing prevalence of thyroid disorders with advancing age, but this is most pertinent with hyperthyroidism. This explains the high percentage of patients with hyperthyroidism in our group 3. Nevertheless excluding patients with hyperthyroidism still showed a significant difference in the insulin-free period between the groups.

## Conclusion

Our retrospective study suggests an interaction of thyroid diseases with metabolic control in type 2 diabetes resulting in an earlier need of insulin treatment. Patients with compensated as well as newly diagnosed thyroid disorders required significantly earlier insulin therapy compared to patients without thyroid diseases at the time of diabetes onset. These data reinforce that diabetes patients with thyroid comorbidity need more endocrine attention. Due to the retrospective design, the limited number of patients and the high percentage of missing data in some patient groups our results need to be interpreted with caution. Further studies are needed to confirm our findings and elucidate mechanisms of interaction of thyroid disease in type 2 diabetes patients.

## Abbreviations

anti-GAD: Autoantibodies against glutamate decarboxylase; anti-TPO: Thyroid peroxidase; anti-Tg: Thyroglobulin; TRAb: Thyrotropin receptor; fT3: Free triiodothyronine; fT4: Free thyroxine; TSH: Thyroid-stimulating hormone; AITD: Autoimmune thyroid disease; AFT: Autonomously functioning thyroid; BMI: Body mass index; Q1: First quartile; Q3: Third quartile; rs: Correlation coefficient; rs2: Coefficient of determination; LADA: Latent autoimmune diabetes in adults.

## Competing interests

The authors declare that they have no competing interests.

## Authors’ contribution

VW, DS and KB contributed to conception and design, acquisition of data, analysis and interpretation of data; DB has been involved in drafting the manuscript and revising it critically for important intellectual content. All authors read and approved the final manuscript.
